# Aging services workers in the pandemic: Voiced experience of senior center staff & case workers

**DOI:** 10.1093/geroni/igab046.3462

**Published:** 2021-12-17

**Authors:** Esther Okang, Siobhan Aaron, Katherine Supiano, Abdul Osman

**Affiliations:** 1 University of Utah, Salt Lake City, Utah, United States; 2 University of Utah, Salt Lke City, Utah, United States

## Abstract

The pandemic necessitated immediate shutdown of senior centers, requiring a rapid pivot in the delivery of services to older adults by direct care workers. We provided psychosocial support to older adult service personnel-including Aging and Adult Services case workers and Senior Center Staff, and conducted focus groups with staff at intervals to capture the mid-point of the pandemic (peak of older adult deaths), onset of vaccine availability and the re-entry phase as programs re-opened. We evaluated coping and self-efficacy of workers and discerned sustained high levels of coping and perceived job performance. Using a phenomenological lens, we analyzed transcribed recordings, generated codes, and created categories of experiences. Several themes emerged: personal and professional resilience, passion for serving older adults, motivation to perform their job well, stress of not having face-to-face contact with clients, insufficient resources-especially in rural areas, lack of essential training, feeling disjointed as a team, and work-life balance. Over the course of the pandemic, workers expressed increasing resiliency and skills to navigate the pandemic, oscillations in their fears for their clients’ well-being, and gratitude that they kept their jobs and gained additional State resources. As the vaccine was available and utilized, and as senior centers were reopening, senior center staff were enthusiastic, yet case workers remained apprehensive about long-term consequences of the pandemic. This study affirms the role of direct care workers as essential and valuable. Yet, their expressed need for more education, psychosocial support, and community awareness of their service remains to be addressed.

